# Extracellular vesicles derived from lung cancer cells exposed to intermittent hypoxia upregulate programmed death ligand 1 expression in macrophages

**DOI:** 10.1007/s11325-021-02369-1

**Published:** 2021-07-12

**Authors:** Yuanling Liu, Minzhen Lu, Jianan Chen, Siqi Li, Yiyu Deng, Shifang Yang, Qiong Ou, Jing Li, Ping Gao, Zeru Luo, Ping Yuan, Jianlong Tan, Xinglin Gao

**Affiliations:** 1 The Second School of Clinical Medicine, Southern Medical University, Guangzhou, 510515 China; 2 Department of Pulmonary and Critical Care Medicine, Guangdong Provincial People’s Hospital, Guangdong Academy of Medical Sciences, Zhongshan 2nd road No. 106, Guangzhou, 510080 China; 3 Guangdong Provincial Geriatrics Institute, Guangzhou, 510080 China; 4 Department of Critical Care and Emergency, Guangdong Provincial People’s Hospital, Guangdong Academy of Medical Sciences, Guangzhou, 510080 China

**Keywords:** Obstructive sleep apnea, Non-small-cell lung cancer, Extracellular vesicle, Programmed cell death-1 ligand, Macrophage

## Abstract

**Purpose:**

Intermittent hypoxia (IH), a hallmark of obstructive sleep apnea (OSA), compromises immune surveillance through the upregulation of programmed cell death-1 ligand (PD-L1). Tumor-released extracellular vesicles (EVs) have been reported to modulate immunosuppressive activities. We investigated whether or not EVs derived from intermittent hypoxic lung cancer cells can alter the expression of PD-L1 in macrophages.

**Methods:**

The expression of PD-L1^+^monocytes from 40 patients with newly diagnosed non-small-cell lung cancer (NSCLC) and with (*n*=21) or without (*n*=19) OSA were detected. Plasma EVs isolated from NSCLC patients with moderate–severe OSA (*n*=4) and without OSA (*n*=4) were co-cultured with macrophages. A549 cells were exposed to normoxia or IH (48 cycles of 5 min of 1% O_2_ hypoxia, followed by 5 min of normoxia). EVs were isolated from cell supernatant and were co-cultured with macrophages differentiated from THP-1. PD-L1 and hypoxia-inducible factor-1 α (HIF-1α) expressions were measured by flow cytometry, immunofluorescence, and Western blot analysis.

**Results:**

PD-L1^+^monocytes were elevated in NSCLC patients with OSA and increased with the severity of OSA and nocturnal desaturation. PD-L1^+^ macrophages were induced by EVs from NSCLC patients with OSA and positively correlated with HIF-1α expressions. EVs from IH-treated A549 can promote PD-L1 and HIF-1α expression in macrophages and the upregulation of PD-L1 expression was reversed by specific HIF-1α inhibitor.

**Conclusion:**

IH can enhance the function of EVs derived from lung cancer cells to aggravate immunosuppressive status in macrophages. HIF-1α may play an important role in this process.

**Supplementary Information:**

The online version contains supplementary material available at 10.1007/s11325-021-02369-1.

## Introduction

Obstructive sleep apnea (OSA) is a relatively common disease characterized by recurrent total or partial upper airway collapse during sleep. This is related with intermittent hypoxia (IH) and sleep fragmentation. Some large cohort studies have found evidences that OSA promotes cancer development and increases cancer mortality [1–4]. It has been proposed that hypoxia-induced immune deregulation might be the potential mechanism of adverse prognosis of cancer patients complicated with OSA [1]. Some in vivo and in vitro studies show that intermittent hypoxia influences cancer cell behavior and promotes lung cancer metastasis [5–7]. Marta Torres et al. found that IH promoted lung cancer aggressiveness through alterations in the host immune response in a murine model of OSA [8].

The inhibitory molecules’ programmed cell death receptor 1 (PD-1) and its ligand (PD-L1) suppress anti-cancer immunity. PD-L1 on monocytes or macrophages binds to PD-1 expressed on T-cells, B-cells, dendritic cells, and natural killer T-cells, which would inhibit signaling pathways that activate T-cells [9]. Therefore, anti-PD-L1 and anti-PD-1 antibodies have been used for cancer immunotherapy. Huang et al. showed that intermittent hypoxia enhanced the tumor PD-L1 expression in a mouse model of OSA [10]. In OSA patients, intermittent hypoxia could upregulate the expression of PD-L1/PD-1 on monocytes as a result of hypoxia-inducible factor-1 α (HIF-1α) activation [11, 12]. HIF-1α is the first and widely known signaling molecule for intermittent hypoxia. HIF-1α binds to a hypoxia response element (HRE) of the PD-L1 promoter and activates PD-L1 transcription [13]. However, the PD-L1/PD-1 axis in lung cancer patients with OSA is still poorly understood.

Cancer cell-derived extracellular vesicles (EVs) play an important role in intercellular communication between tumor cells and immune cells in local and distant microenvironments [14]. Tumor-secreted EVs are reported to promote PD-L1 expressions in monocytes or macrophages [15, 16]. EVs include several categories: exosomes (30–100 nm diameter), which are derived from multi-vesicular bodies or from the plasma membrane; microvesicles (100–1000 nm diameter); and apoptotic bodies (>1 μm diameter), which are generated from dying cells [17, 18]. EVs contain bioactive proteins, lipids, and nucleic acids and can be detected in blood, urine, and other body fluid, which has the unique potential to be a biomarker [19]. Recent study has found that IH-induced circulating exosomes enhance tumor cell malignant properties [20]. It has been reported that intermittent hypoxia and the cancer cell-derived exosomes can promote PD-L1 expression in monocytes or macrophages.

Based on aforementioned considerations, we hypothesized that IH may enhance the function of EVs derived from cancer cells to upregulate PD-L1 expression in macrophages. Hence, the objective of the present study was to analyze potential associations between the PD-L1^+^monocytes expression and OSA in lung cancer patients, and confirm that EVs isolated from lung cancer patients with severe OSA or lung cancer cells treated with IH upregulate PD-L1 expression in macrophages.

## Material and methods

### Participants and blood samples

From April 2019 to May 2020, 40 newly diagnosed non-small-cell lung cancer (NSCLC) patients aged 40–76 years were recruited from the Department of Respiratory and Critical Care Medicine of Guangdong Provincial People’s Hospital, Guangzhou, and were divided into group NSCLC (*n*=19) and group OSA+NSCLC (*n*=21) according to OSA diagnostic testing. All participants underwent overnight sleep testing (The Alice Night One, Philips Respironics, Inc., Murrysville, PA, USA) in the hospital. Oronasal flow and pressure, heart rate, thoracic and abdominal respiratory movements, and arterial oxygen saturation (SpO_2_) were recorded. Oxygen desaturation index (ODI) was defined as the number of falls of oxygen saturation ≥4% per hour. OSA was defined as apnea-hypopnea index (AHI) ≥5 events/h according to the American Academy of Sleep Medicine 2012 Task Force [21]. All patients were diagnosed with NSCLC by histological pathology. NSCLC stages were categorized according to Union for International Cancer Control (UICC) TNM staging system officially promulgated in January 2017 [22].

Exclusion criteria include the use of oral appliance, oxygen supplement, drugs that affect blood coagulation, received anti-tumor therapy; history of chronic airway diseases, respiratory failure, allergic disease, any infectious disease in the previous 3 months, severe mental disorders, pulmonary resection, stroke, autoimmune diseases dyslipidemia; and diagnosis of cancers except for NSCLC.

**Fig. 1:**
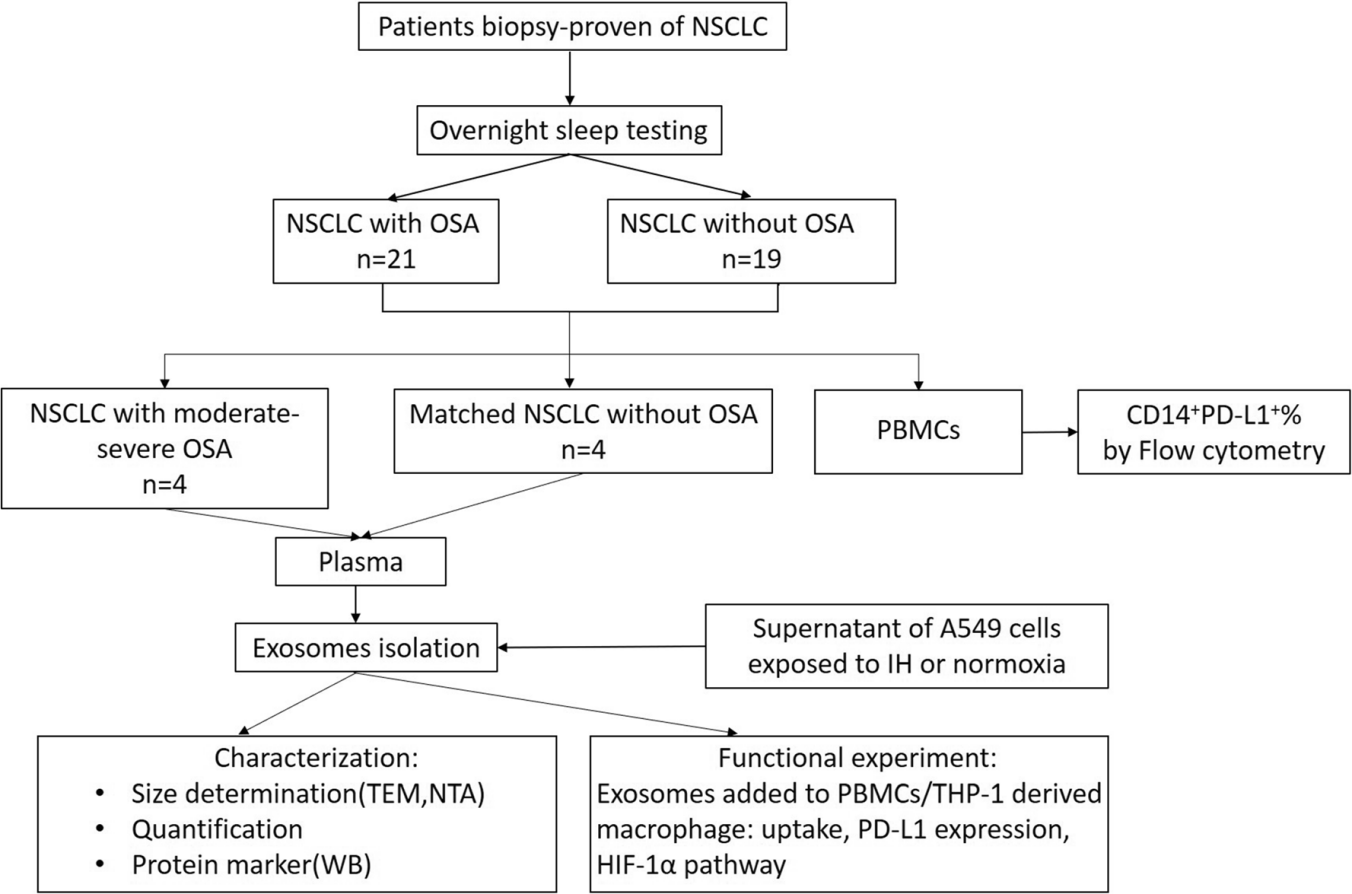
Subject recruitment and experimental design. OSA, obstructive sleep apnea; NSCLC, non-small-cell lung cancer; PBMCs, peripheral blood mononuclear cells; PD-L1, programmed cell death receptor ligand 1; TEM, transmission electron microscopy; NTA, nanoparticle tracking analysis; IH, intermittent hypoxia; WB, Western blot; HIF-1α, hypoxia-inducible factor alpha

Peripheral blood was collected from all participants. Healthy donor peripheral blood was obtained in physical examination center of Guangdong Provincial People’s Hospital between April 2019 and May 2020. PBMCs isolated from the group NSCLC and group OSA+NSCLC were used to measure the percentages of PD-L1^+^monocytes by flow cytometry. Plasma isolated from NSCLC patients with moderate-severe OSA (group N+O, *n*=4) and clinically matched NSCLC patients (group N, *n*=4) was used for EV isolation. PBMCs isolated from healthy donor were collected for T-cell function experiment and differentiated into macrophage. The experimental design is shown in Fig. 1.

The study was approved by the Ethics Committee of Guangdong Provincial People’s Hospital (No. GDREC2017259H(R1)). All patients gave written informed consent.

### Cell culture and IH model

PBMCs were isolated using Ficoll-400 (Sigma-Aldrich) according to manufacturer’s instructions. ACK Lysis Buffer (Sigma-Aldrich) was added to the collected cells to lyse the blood cells. PBMCs were washed 3 times by phosphate buffer saline (PBS, Gibco, USA) and cultured in Roswell Park Memorial Institute 1640 (RPMI 1640, Gibco, USA) supplemented with 10% fetal bovine serum (FBS, Gibco, USA) at 37°C with 5% CO_2_. PBMCs were differentiated into macrophages using 20 ng/mL human M-CSF (Sigma) for 6 days. The media were changed at day 3.

The adenocarcinoma cell line A549 and human monocytic THP-1 cells (Cell Bank of the Chinese Academy of Sciences, Shanghai, China) were cultured in RPMI 1640 (Gibco, USA) containing 10% fetal bovine serum, penicillin (100U/mL), and streptomycin (100mg/mL). THP-1 monocytes were differentiated into macrophages (mTHP-1) using 100 ng/mL phorbol 12-myristate 13-acetate (PMA, Sigma, P8139) at 37°C with 5% CO_2_ for 48 h.

When A549 cells were 70–80% confluent, they were detached by trypsin-EDTA and passaged at a ratio of 1:3 and the third-fifth passage A549 cells were used for exosome isolation and IH treatment. The A549 cells were divided into normoxia (NA) group and IH group. The A549 cells in NA group were cultured at 37°C with 5% CO_2_ and 21% O_2_. Hypoxia condition was performed using Modular Incubator Chambers (Billups Rothenberg Inc., San Diego, CA) with an O_2_ Quickstick Oxygen Analyzer (Nuvair, CA93033, USA) (Fig. S1A). For IH, the chamber was flushed with 1% O_2_, 5% CO_2_, and 94% N_2_ pressurized gas (Guang Qi Gas, Guangzhou, China) until the oxygen concentration reduced to 1% and the chamber was maintained at 37°C with 1% O_2_ for 5 min. Then, the cover of the chamber was removed in room air until the oxygen concentration reached to 21% and the chamber was maintained at 37°C with 21% O_2_ for 5 min. The IH group was exposed to 48 cycles of 5 min of hypoxia followed by 5 min of normoxia (Fig. S1B).

### EV isolation and quantification

Plasma was isolated from peripheral blood samples using centrifugation at 2000*g* for 20 min at 4°C and stored at −80°C until further analysis. As previous study described [23], EVs were isolated from plasma using ExoQuick plasma prep and exosome precipitation kit (EXOQ5TM-1, System Biosciences) according to the manufacturer’s protocol. Briefly, 3 μL of (611U/mL) thrombin was added to 300-μL plasma and incubated at room temperature for 5 min. After centrifugation at 10,000 rpm for 5 min at 4°C, about 250-μL supernatant was collected. Then, the supernatant was centrifuged at 3000*g* for 15 min at 4°C and then transferred to a sterile vessel. Sixty-three-microliter ExoQuick Exosome Precipitation Solution was added to the bio-fluid, mixed well, and refrigerated 30 min at 4°C. Then, the ExoQuick/biofluid mixture was centrifuged twice at 1500g for 30 min. The exosome pellet was resuspended in 100-μL PBS. The EVs isolated from plasma of NSCLC patients with and without OSA are designated respectively as EV-N+O and EV-N for simplicity.

Before EV isolation, A549 cells were plated at 10-cm plates and cultured in RPMI 1640 medium with 10% exosome-free fetal bovine serum (FBS, System Biosciences) for 48h. From the twelfth hour, 12 plates of A549 cells were exposed to 48 cycles of IH, and the other 12 plates of A549 cells were cultured in normoxia. For vesicle enrichment [24], media were pooled and centrifuged at 300*g* for 10 min, 2000*g* for 20 min, and 10,000*g* for 30 min at 4°C, followed by filtration using a 0.22-μm filter; the supernatant was then centrifuged at 100,000*g* for 90 min at 4°C, and the EV precipitate was washed using PBS by centrifuging at 100,000*g* for 90 min at 4°C. The EV pellet was resuspended in 400-μL PBS. The EVs isolated from NA group and IH group are designated as EV-NA and EV-IH for simplicity, respectively.

EVs were quantified by a protein assay (BCA™ Protein Assay Kit, Pierce, USA) as previously described [25]. Markers of exosomes, including CD9 and tumor susceptibility gene 101 (TSG101), were analyzed by Western blot.

### Transmission electron microscopy

Approximately, 10 μL of EVs were placed on 200-mesh copper grids, and incubated for 3 min at room temperature. Then, EVs were negatively stained with 3% phosphotungstic acid (pH=7.0) for 3 min at room temperature. The grids were washed by pure water, and air-dried. EVs were observed through TEM (Hitachi H-7650, Hitachi, Japan).

### Nanoparticle tracking analysis

NTA was performed with the NanoSight NS300 (Malvern Instruments, Ltd., Malvern, UK). Purified EVs were diluted 10- to 100-fold in PBS to measure the particle size and concentration. The software, NTA 3.3 Dev Build 3.3.301 (Malvern Instruments Ltd.), was used to analyze the data. Capture settings were as follows: camera type, sCMOS; laser type, Blue488; camera level, 16; slider shutter, 1300; slider gain, 512; FPS, 25.0; analysis settings, detect threshold, 5; blur size, auto; max jump distance, 13.2–13.8 pix. Sample readings were taken in triplicate over 30s at 10 frames per second at room temperature. Exosome concentration analysis was normalized with the volume of plasma or the total number of cells from the corresponding dish. The total number of cells was counted by cell-count boards.

### EV treatment

PBMCs isolated from healthy donor or THP-1 were seeded into 6-well plates with density of 2×10^5^ per well and differentiated into macrophages before treatment. A total of 10μg/mL EVs were directly added into culture medium (2mL culture medium per well). PBS was added as control. After 48h, cells were harvested for the following experiments.

### HIF-1α inhibition

mTHP-1 was seeded into 6-well plates with density of 2×10^5^ per well and co-cultured with a HIF-1α inhibitor (BAY87-2243, 10μM, Selleck Company, USA) for 48 h.

### Immunofluorescence assay

#### Cellular internalization of exosomes

Purified EVs were labeled with a PKH26 red fluorescent labeling kit (Sigma-Aldrich, USA) according to the manufacturer’s instructions. Briefly, EVs were resuspended with 1-mL Diluent C. Four-microliter PKH26 dye was diluted with 1-mL Diluent C as working solution. Then, EVs were cultured with working solution (1:1) at room temperature for 5 min, then washed by exosome-free FBS/RPMI-1640 and PBS. PKH26-labeled EVs were isolated by ultracentrifugation at 100,000*g* for 90 min at 4 °C. A mixture without EVs was used as the negative control. Then, PKH26-labeled EVs (10μg/mL) were co-cultured with PBMC-derived macrophages or mTHP-1 for 12 h. Then, the cells were incubated in 4% paraformaldehyde for 20 min, permeabilized with 0.1% Triton X-100, and stained with DAPI.

#### PD-L1 immunofluorescence assay

Cells were seeded into confocal dish and fixed by 4% paraformaldehyde for 20 min. Then, cells were washed 3 times, permeabilized with 0.1% Triton X-100, and blocked with 5% normal goat serum for 30 min at room temperature. Next, cells were incubated with anti-PD-L1 primary antibody (ab213524, 1:100, Abcam, Cambridge, UK) over night at 4°C, and then incubated with Alexa Fluor 488 Goat Anti-Rabbit IgG secondary antibody (ab150077, 1:1000, Abcam, Cambridge, UK) for 1 h at room temperature in a dark chamber. The nuclear was stained with DAPI.

Finally, the cells were observed under a laser scanning confocal microscope (Leica SP5-FCS, German). Images were analyzed using ImageJ software.

### Flow cytometry

PBMCs isolated from NSCLC patients with and without OSA were incubated with Fc Receptor Binding Inhibitor Antibody (Invitrogen eBiosciences) for 20 min, then were stained with Alexa 674 conjugated anti-human CD14 antibodies (BD Biosciences) and PE-cy7 conjugated antihuman PD-L1 antibodies (BD Biosciences). PBMC-derived macrophages or mTHP-1 treated with EVs were detached from the 6-well plates using ice-cold PBS. The cells were collected and stained with LIVE/DEAD dye (LIVE/DEAD™ Fixable Dead Cell Stain Sampler Kit, Thermo Fisher Scientific), followed by PE-cy7 conjugated antihuman PD-L1 antibodies (BD Biosciences). Then cells were permeabilized using Intracellular Fixation kit (Thermo Fisher Scientific) and stained with Alexa 674 conjugated anti-human CD68 antibodies (BD Biosciences) according to a standard flow cytometry staining protocol. Mouse IgG1-PE-cy7 antibodies were used as isotype controls.

After staining, the cells were washed twice and re-suspended using PBS for immunophenotypic analysis. All analyses were performed using FACS Calibur (BD Biosciences). Data were analyzed with FlowJo software (Tree Star, Inc.). Phenotypic analyses are showed in Fig. S2.

### Western blot analysis

The protein lysate used for Western blotting was extracted using RIPA Lysis (Thermo Fisher Scientific) containing protease inhibitors (BOSTER Biological, China). Proteins were quantified using the BCA™ Protein Assay Kit (Pierce, USA). Thirty micrograms of total cell lysate or EVs were separated on 10% gels by SDS-PAGE and electrophoretically transferred to polyvinylidene difluoride membranes (Millipore, Danvers, MA, USA) using a Bio-Rad Bis-Tris Gel system (Bio-Rad, CA, USA). Membranes were probed using primary antibodies against PD-L1 (ab213524, 1:1000), CD9 (ab92726, 1:1000), TSG101 (ab125011, 1:1000), and HIF-1α (ab51608, 1:1000, all from Abcam, Cambridge, UK); α-Tubulin (#2144, 1:1000, Cell Signaling Technology). Membranes were then incubated with a horseradish peroxidase-conjugated secondary antibody. The chemiluminescence reagent (Millipore, Danvers, MA, USA) was used to visualize the immunoreactive bands and signals were obtained from an Image Quant™ LAS-500 Mini Imager (Fuji, Japan). The protein density of each band was determined using ImageJ software.

### Statistical analysis

**Fig. 2:**
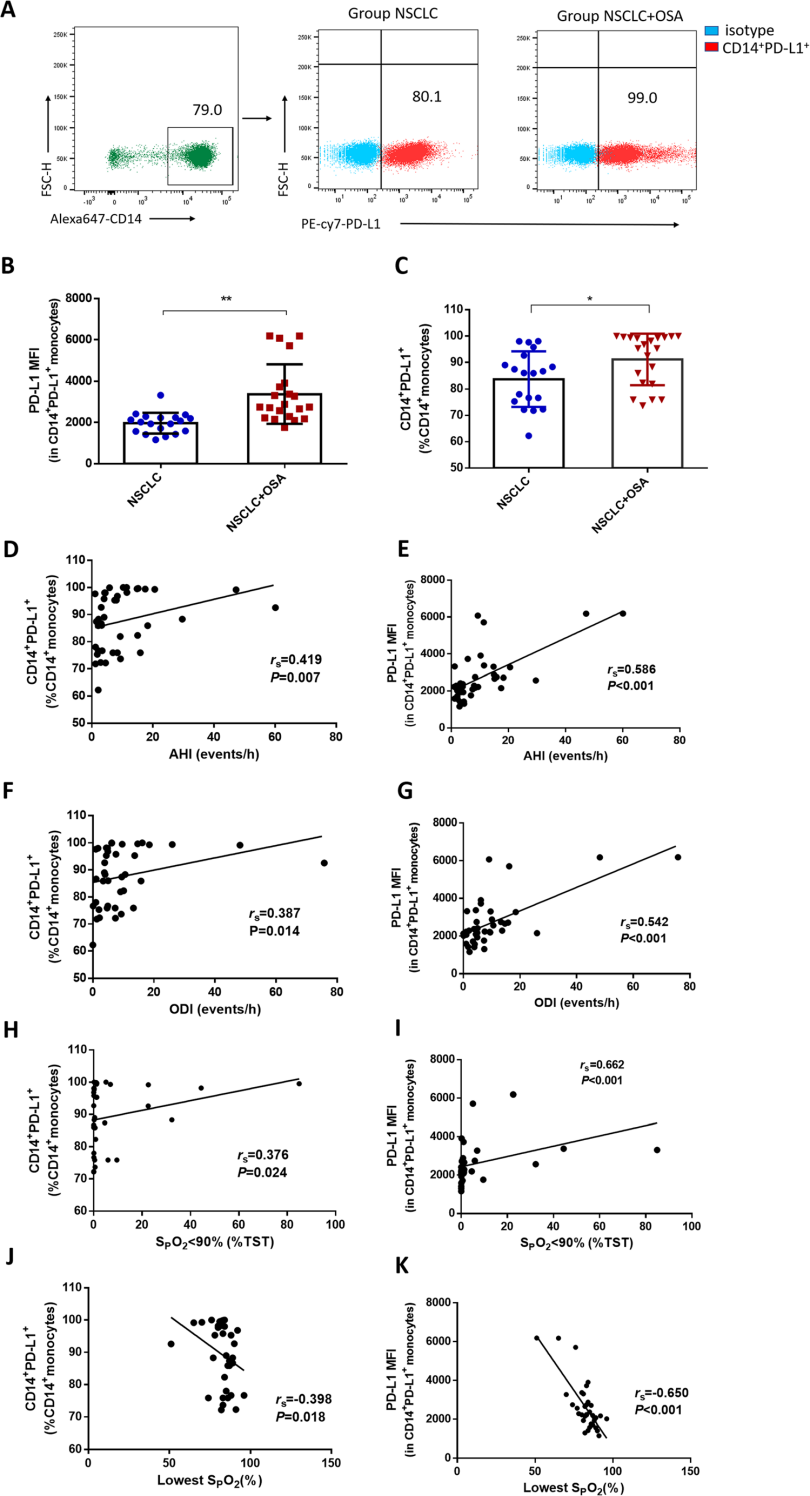
Programmed cell death ligand-1 (PD-L1) was overexpressed on monocytes from non-small-cell lung cancer (NSCLC) patients with obstructive sleep apnea (OSA). **a** The representative flow cytometry analysis of CD14^+^PD-L1^+^ expression on peripheral blood PBMCs from NSCLC patients with and without OSA. **b**–**c** The percentage of PD-L1^+^monocytes and PD-L1 MFI were higher in NSCLC group with OSA than in NSCLC+OSA group. The percentage of PD-L1^+^monocytes and PD-L1 MFI were positively correlated with AHI (**d**, **e**), ODI (**f**, **g**), and the recording time with SpO_2_<90% (**h**, **i**). The percentage of PD-L1^+^monocytes and PD-L1 MFI were related with the lowest SpO_2_ (**j**, **k**). *P <0.05, **P < 0.01

**Fig. 3:**
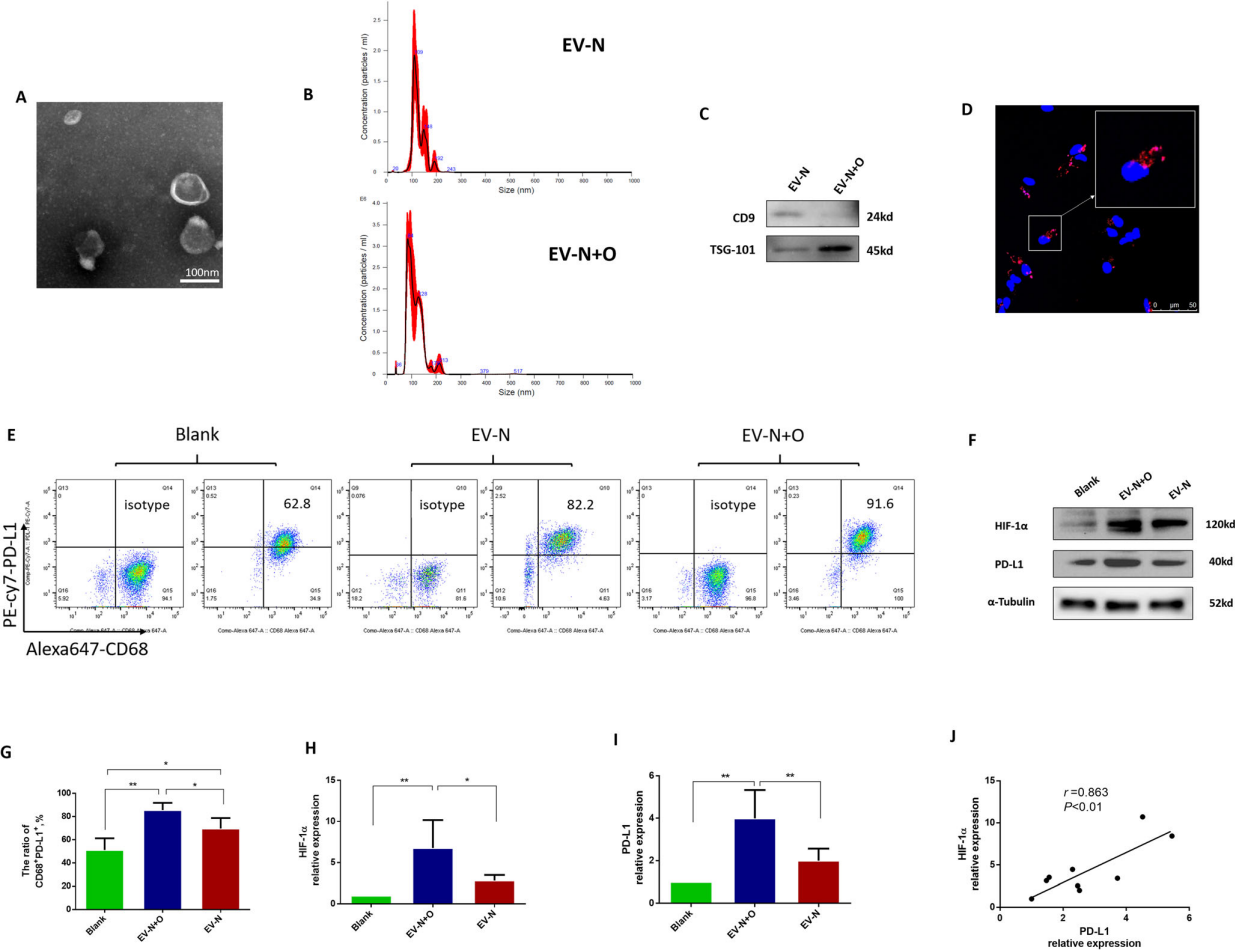
EVs isolated from NSCLC patients with OSA promoted PD-L1 and HIF-1α expression in macrophages. **a**–**c** Characterization of EVs isolated from plasma of NSCLC patients. **a** Transmission electron microscope (TEM) images of exosomes. **b** Freshly isolated EVs were diluted 1:100 for NTA technology. The curve of the graph illustrated that the majority of EV-N and EV-N+O were distributed with a peak at size 109 nm and 84 nm, respectively. **c** Western blot analysis showed that CD9 and TSG 101 were expressed in EVs. **d** Uptake of EVs by THP-1-derived macrophages. EVs were labeled with PKH26 (red), and nuclei with DAPI (blue). **e**–**j** Macrophages differentiated from PBMCs respectively were co-cultured with EV-N, EV-N+O, and PBS (blank) for 48 h before evaluation of PD-L1 and HIF-1α expression by flow cytometry and western blot analysis. **e**, **g** EV-N+O promoted the expression of CD68^+^PD-L1^+^ macrophages. **f**, **h**, **i** EVs isolated from NSCLC patients with OSA promote PD-L1 and HIF-1α expression in macrophages. **j** The positive correlation between HIF-1α and PD-L1 expressions. Data are expressed as the mean ± SEM. *P <0.05, **P < 0.01

**Fig. 4:**
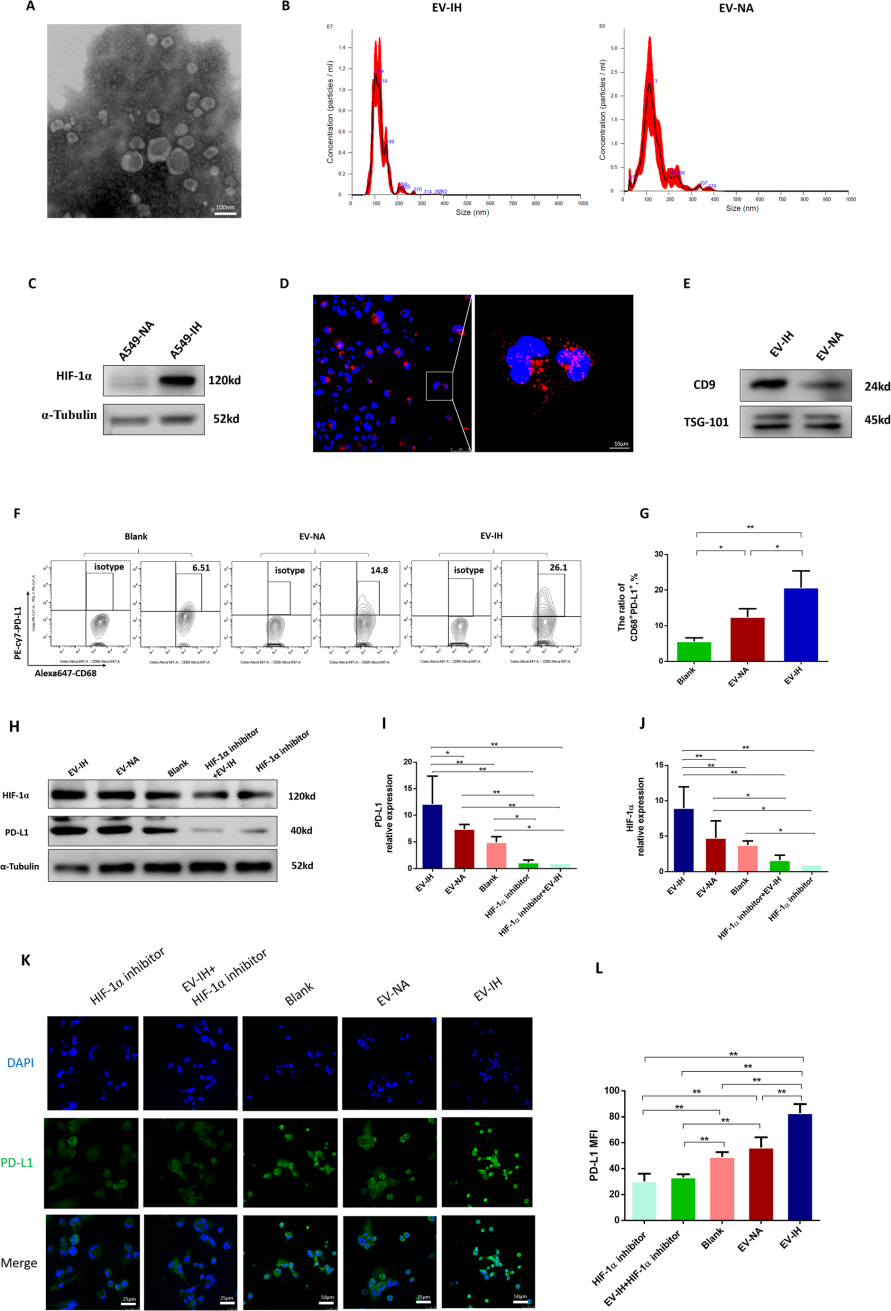
EV-IH regulated PD-L1 expression in macrophages in vitro through HIF-1α pathway. **a** Transmission electron microscope (TEM) images of EVs isolated from supernatant samples of A549 cells. **b** Freshly isolated EVs were diluted 1:100 for nanoparticle tracking analysis using nanosight technology. The curve of the graph illustrated that the majority of EV-IH and EV-NA were distributed with a peak at size 104 nm and113 nm, respectively. **c** Western blot analysis showed that HIF-1α was overexpressed in IH-treated A549 cells. **d** Uptake of EVs by THP-1-derived macrophages. EVs were labeled with PKH26 (red), and nuclei with DAPI (blue). **e** CD9 and TSG 101 expression in EVs were assessed by Western blot analysis. Macrophages differentiated from THP-1 cells (mTHP-1) respectively were co-cultured with EV-NA, EV-IH, BAY87-2243(HIF-1α inhibitor, 10μM), EV-IH (10 ug/mL) combined with BAY87-2243, and PBS (blank) for 48 h before evaluation of PD-L1 and HIF-1α expression by flow cytometry, immunofluorescence, and Western blot analysis. **f**–**g** EV-IH promoted the expression of CD68^+^PD-L1^+^ macrophages. **h**–**j** Western blot analysis showed that EVs from IH treated A549 cells can upregulate PD-L1 and HIF-1α expression in macrophages. Specific HIF-1α inhibitor BAY87-2243 inhibited the upregulation of PD-L1 expression in the EV-IH group. **k**–**l** Immunofluorescence was employed to locate PD-L1. Both cell membrane and cytoplasm in mTHP-1 showed PD-L1 signal (green fluorescence). The mean fluorescence intensity (MFI) levels of PD-L1 in different groups were matched with Western blot analyses. Data are expressed as the mean ± SEM. *P <0.05, **P < 0.01

Normal distribution data were presented as mean ± standard deviation (SD), and non-normal distribution data were presented as median (inter-quartile range [IQR]). Fisher’s exact tests and chi-squared test were used to analyze categorical data in Table 1 and Table S1. Comparisons of clinic characteristics in Table 1 and Table S1 between two groups were performed by Student’s *t* tests or Mann-Whitney *U* tests. The data in Fig. 2b were compared using Student’s *t* tests after logarithm transition. The data in Fig. 2c were compared using Mann-Whitney *U* tests because of non-normal distribution. The correlations between the data in Fig. 2d–2k were assessed with Spearman’s rank correlation because of non-normal distribution. The correlations between the data in Fig. 3j were compared using Pearson correlation. One-way ANOVA with LSD *t* tests for multiple comparisons were employed to compare the data in Figs. 3 and 4 because of total variance homogeneity. All statistics were two-sided. *P*<0.05 was considered as statistical significance. SPSS statistical software (version 22.0, Chicago, IL) was used for all data analyses.

**Table 1:** Clinic characteristics of subjects in the study

Parameter	NSCLC *n*=19	NSCLC+OSA *n*=21	*P* value
Age (years)	61.26±11.58	61.90±8.58	0.842 ^a^
Male proportion	11 (58)	15 (71)	0.370 ^b^
BMI (kg/m^2^)	23.21±2.45	23.49±3.26	0.813 ^a^
Smoking proportion	5 (26)	9 (43)	0.273 ^b^
Medical history			
Coronary heart disease	2	2	1.000 ^e^
Hypertension	1	2	1.000 ^e^
Diabetes	2	2	1.000 ^e^
Histology			0.906 ^b^
Squamous cell carcinoma	5	9	
Adenocarcinoma	14	12	
Pathological stage			0.970^e^
I	5	4	
II	3	3	
III	5	7	
IV	6	7	
ESS	2.50 (0.00, 7.00)	8.00 (7.00, 9.50)	<0.001 ^d^
AHI (events/h)	3.00 (2.10, 4.00)	13.15 (8.40, 18.10)	<0.001 ^d^
5–15 (*n*)	0	13	
16–30 (*n*)	0	6	
>30 (*n*)	0	2	
ODI (events/h)	3.60(1.03,4.88)	10.35(6.20,16.10)	<0.001 ^d^
SpO_2_ <90% (% TRT)	0.10(0.00,0.20)	1.30(0.65,19.28)	<0.001 ^c^
Lowest SpO_2_ (%)	86.00(82.25–89.00)	82.00(76.25,84.00)	0.015 ^c^
85%–90% (*n*)	14	6	
80%–84% (*n*)	5	8	
<80% (*n*)	0	7	

Data are presented as *n*, *n* (%), median (IQR), or mean ± SD

*OSA*, obstructive sleep apnea; *NSCLC*, non-small-cell lung cancer; *ESS*, Epworth sleepiness scale; *AHI*, apnea hypopnea index; *ODI*, oxygen desaturation index; *TRT*, total recording time

^a^*t* tests

^b^Chi-squared test

^c^Mann-Whitney *U* tests

^d^*t* tests after logarithm transition

^e^Fisher’s exact tests

## Results

### Patient characteristics

Between group NSCLC and group NACLC+OSA, the age, gender, body mass index (BMI), smoking proportion, pathological stage, histology, and medical history did not significantly differ (*P*>0.05). Patients in group NACLC+OSA had higher AHI, oxygen desaturation index (ODI), Epworth sleepiness scale (ESS) scores, SpO_2_ <90% of total recording time (TRT), and lower oxygen saturation nadir than that in group NSCLC (Table 1). There were no significant differences in group N+O and group N regarding to age, gender, BMI, smoking proportion, pathological stage, histology, and medical history (*P*>0.05). The AHI, ODI, SpO_2_<90% (%TRT), ESS scores, and the mean fluorescence intensity (MFI) of PD-L1 were higher in group N+O than in group N. The lowest SpO_2_ in group N+O was lower than that in group N (*P*<0.05). The percentages of PD-L1^+^monocytes were of no significant differences between two groups (*P*>0.05) (Table S1).

### PD-L1 was overexpressed in monocytes from NSCLC patients with OSA

The percentages of PD-L1^+^monocytes and PD-L1 MFI were higher in NSCLC patients with OSA than in the NSCLC patients without OSA (PD-L1^+^monocytes 86.00 (75.30%, 97.70%) vs. 95.30% (82.10%, 99.45%), *P*=0.017, Fig. 2c; PD-L1 MFI 2025 (1569, 2246) vs. 2753 (2255, 3819), *P*<0.001, Fig. 2b). Interestingly, the percentages of PD-L1^+^monocytes and PD-L1 MFI were positively correlated with AHI (Fig. 2d, 2e), ODI (Fig. 2f, 2g), and SpO_2_ <90% (%TRT) (Fig. 2h, 2i). In addition, the percentages of PD-L1^+^ monocytes were negatively correlated with the lowest SpO_2_ (Fig. 2j, 2k).

### EVs isolated from NSCLC patients with moderate–severe OSA promoted PD-L1 and HIF-1α expression in macrophage

To study the effects of EVs isolated from NSCLC patients with and without OSA on the PD-L1 expression of macrophage, we first investigated the characterization of EVs. Negative stain transmission electron image showed vesicles in typically shaped morphology (Fig. 3a). The average diameter of EV-N+O was 113.9±4.0nm, while EV-N was 126.6±4.8 nm (Fig. 3b). EV concentrations in group N+O was significantly higher than that in group N after normalization with equal volume of plasma (1.71×10^10^±1.95×10^9^ vs. 7.16×10^9^±1.32×10^9^ particles/ml, *P*<0.05, Fig. 3b). The EV protein concentration in EV-N+O was significantly higher than that in EV-N (2.50±0.64 vs. 1.04±0.19μg/μL, *P*<0.05). Western blot analysis revealed the presence of characteristic exosomal maker CD9 and TSG101 [26] (Fig. 3c).

To examine whether EVs could be taken up by macrophages, PKH26 (Red)-labeled EVs were added to PBMC-derived macrophages and incubated for 12h. As shown in Fig. 3d, red fluorescence was clearly observed around the DAPI-labeled nuclei in macrophages, which supported that the extracellular EVs could be taken up by PBMC-derived macrophages.

To assess the efficacy of EV-N or EV-N+O on macrophages, PBMC-derived macrophages were treated with EVs and PBS, and were collected after 48h. Flow cytometry analysis showed that CD68^+^PD-L1^+^ macrophages were significantly higher in group EV-N+O and group EV-N than in group blank (Fig. 3e, g). The proportion of CD68^+^PD-L1^+^ macrophages in EV-N+O group was higher than that in EV-N group (Fig. 3e, g). To determine whether these EVs regulate PD-L1 and HIF-1α expression, cellular HIF-1α and PD-L1 protein levels were assessed by Western blot analysis. EV-N+O or EV-N treatment could upregulate the HIF-1α and PD-L1 expressions in macrophages (Fig. 3f, h, i). Interestingly, both HIF-1α and PD-L1 expressions were higher in group EV-N+O than in group EV-N (Fig. 3f, h, i). Figure 3j shows the positive correlation between HIF-1α and PD-L1 expressions.

### EVs derived from intermittent hypoxic lung cancer cells upregulated PD-L1 in mTHP-1 through HIF-1α pathway

To determine if IH promotes the expression of PD-L1 in macrophages through lung cancer cell-derived EVs, we exposed A549 cells to IH (Fig. S1A, S1B) or normoxia. The expression of HIF-1α in A549 increased under IH condition (Fig. 4c). EVs were isolated from cell culture supernatant through sequential ultracentrifugation. Homogeneous populations of typical rounded shape vesicles of EVs were observed from the representative transmission electron image (Fig. 4a). NTA showed an average diameter of 121.9 ± 1.8 nm of the particles in EV-IH and 131.8± 6.7 nm of the particles in EV-NA (Fig. 4b). EV concentrations in group IH was significantly higher than that in group NA after normalization with the total number of cells from the corresponding dish (6.31×10^10^±8.09×10^9^ vs. 1.82×10^10^±5.24×10^9^ particles/ml, *P*<0.05, Fig. 4b). The EVs in EV-IH exhibited higher protein concentration when compared with EV-NA (1.52±0.02 vs. 1.14±0.04μg/μL, *P*<0.01). The Western blot showed that EV-IH and EV-NA were positive for CD9 and TSG101, markers of exosomes (Fig. 4e).

To study the effect of EVs derived from intermittent hypoxic lung cancer cells on mTHP-1, we added the EV-NA and EV-IH to mTHP-1. The mTHP-1 exhibited an efficient uptake of EVs, as indicated by the internalization of PKH26-labeled EVs (Fig. 4d). CD68^+^PD-L1^+^ macrophages were significantly higher in group EV-IH and group EV-NA than in group blank (Fig. 4f, g). The proportion of CD68^+^PD-L1^+^ macrophages in the EV-IH group was higher than that in the Exo-NA group (Fig. 4f, g). To further understand the detailed mechanism underlying the alterations on macrophages with EVs, we examined the HIF-1α pathway. Western blotting demonstrated that the HIF-1α and PD-L1 expressions in mTHP-1were significantly higher in group EV-IH than in group EV-NA and group blank (Fig. 4h, i, j). However, there was no difference between EV-NA group and blank group. The HIF-1α expression was significantly decreased in groups treated with HIF-1α inhibitor (Fig. 4h, i, j). With the addition of the HIF-1α inhibitor, PD-L1 expression in mTHP-1 treated with EV-IH was reduced. Moreover, we employed immunofluorescence to locate the cellular expression of PD-L1. Both cell membrane and cytoplasm in mTHP-1 showed PD-L1 signal (green fluorescence; Fig. 4k). The mean fluorescence intensity (MFI) levels of PD-L1 in different groups were matched with Western blot analyses (Fig. 4k, l).

## Discussion

In the present study, we found that PD-L1^+^monocytes were overexpressed in NSCLC patients with OSA and increased with the severity of OSA and nocturnal desaturation. Then, EVs were isolated from plasma of NSCLC patients with and without OSA and added to macrophages. Interestingly, the expressions of PD-L1 in macrophages were induced by EVs from NSCLC patients with OSA and positively correlated with HIF-1α expressions. Using in vitro IH cell model, we further confirmed that EVs derived from intermittent hypoxic lung cancer cells upregulated PD-L1 in macrophages through HIF-1α pathway. Taken together, our findings suggest that OSA can improve tumor EV function to worsen the immunosuppressive status of macrophages.

Cancer cells could escape from the surveillance of immune system, which is associated with cancer development, progression, and resistance to treatment. The PD-L1/PD-1 immune checkpoint inhibitors have been used as a standard treatment for patients with metastatic NSCLC [27]. The engagement of PD-1 on the T-cell surface with its ligand PD-L1 inhibits T-cell proliferation, induces T-cell apoptosis, and promotes the differentiation of CD4^+^T-cells into regulatory T-cells (Tregs) [28]. Macrophage was an important class of APCs, differentiated from monocytes. It has been reported that PD-L1 was overexpressed on monocytes from OSA patients [12, 29] or cancer patients [30]. However, rare study focused on the immune state of lung cancer patients with and without OSA. In a recent study, the expressions of Foxp3 in Tregs and TGF-β1 in patients with both NSCLC and OSA were elevated, which indicates that OSA may promote maturation and immunosuppressive function of Tregs in NSCLC patients [31]. Here, we found that the expressions of PD-L1 on monocytes were elevated in NSCLC patients with OSA and were positively correlated with the AHI and ODI. This indicates that OSA may aggravate the immunosuppression in macrophages of lung cancer patients. Intermittent hypoxia is one of the most prominent features of OSA. Several experimental reports reveal that PD-L1 expressions on monocytes can be induced by intermittent hypoxia [11, 12, 29]. We found that PD-L1 expressions on monocytes were positively correlated with nocturnal desaturation, which was consistent with the previous research [11]. What we found suggests that IH condition might regulate PD-L1 expression on monocytes in NSCLC patients with OSA.

Recently, EVs have drawn great attention in intercellular communication between cancer cells and immune cells [32]. EVs are membrane vesicles of endocytic origin released by all cells (both healthy and diseased) and the most abundant extracellular vesicle population in peripheral blood [33]. Exosomal cargos including lipids, proteins, DNAs, messenger RNAs, and microRNA can transfer from cell to cell locally and systemically and can be efficiently taken up by macrophages [26]. Tumor cell-derived EVs could deliver immune-stimulatory or immunosuppressive signaling molecules, which regulate the development, maturation, and anti-tumor capacity of targeted immune cells, including T cells, B cells, macrophages, and APCs [26]. Recent study has reported that lung cancer cell-derived exosomes promote M2 macrophage polarization through P53-dependent pathway [34]. Trying to find the link among cancer cells, monocyte-macrophage, and OSA, we compared the effects of EVs on macrophages from NSCLC patients with and without OSA. In our study, both EVs obtained from the plasma of NSCLC patients with and without OSA can upregulate PD-L1 expression in macrophages. Several studies have proved that tumor-derived exosomes could upregulate PD-L1 expressions in monocytes [15, 35, 36]. Surprisingly, the PD-L1 levels in macrophages treated with EVs obtained from the plasma of NSCLC patients with OSA were higher than that with EVs obtained from the plasma of NSCLC patients without OSA. This indicates that OSA may enhance tumor-derived EVs function to upregulate PD-L1 expressions in macrophages from NSCLC patients. Similarly, Almendros et al. [20] has reported that circulating plasma exosomes obtained from OSA mouse model or OSA patients enhances tumor cell line proliferation and migration in vitro. Moreover, we showed that the trend of the expression of the PD-L1 was consistent with HIF-1α level in macrophages treated with EVs. HIF-1α regulates the expression of PD-L1 by binding directly to a transcriptionally active hypoxia-response element (HRE) in the PD-L1 proximal promoter [37, 38]. It has been reported that HIF-1α selectively upregulated PD-L1 on myeloid-derived suppressor cells [38]. These results suggest that OSA may upregulate PD-L1 expressions in macrophages treated with lung cancer cell-derived EVs through HIF-1α pathway.

Knowing these, we creatively used in vitro IH cell model to further explore the mechanism in EV intercellular interaction between cancer cells and macrophages. The patterns of experimental IH have varied greatly across researchers [39]. In moderate to severe OSA patients, IH is characterized by short cycling periods of hypoxia and reoxygenation. We used Modular Incubator Chambers (Billups Rothenberg Inc., San Diego, CA) with an O_2_ Quickstick Oxygen Analyzer (Nuvair, CA93033, USA) to perform IH condition by reference to Ma’s work [40]. Using this system, 6 cycles of normoxia-hypoxia condition can be achieved per hour. This in vitro IH cell model promotes HIF-1α expression in A549 cells, proving the effectiveness of this intervention.

In the present study, PD-L1^+^ macrophages analyzed by flow cytometry were elevated after treated with lung cancer cell-derived EVs, which was consistent with previous literature [15]. In addition, EV-IH upregulate PD-L1 levels in macrophages. However, there were no differences of PD-L1 expressions analyzed by Western blotting (WB) and immunofluorescence (IF) between group EV-NA and group Blank. From the typical images of PD-L1 immunofluorescence, we know that PD-L1 expresses on both cell membrane and cytoplasm. The PD-L1 expressing on cell membrane was measured by flow cytometry, while total PD-L1 protein level was detected by WB and IF. The difference of PD-L1 expression detected by different methods indicates that lung cancer cell-derived EVs mainly influence the expression of PD-L1 on cell membrane; however, EVs derived from intermittent hypoxic lung cancer cells upregulate total PD-L1 protein levels in macrophages. PD-L1^+^ macrophages have been reported to inhibit T cell function and enhance T cells apoptosis [16, 35].

**Fig. 5:**
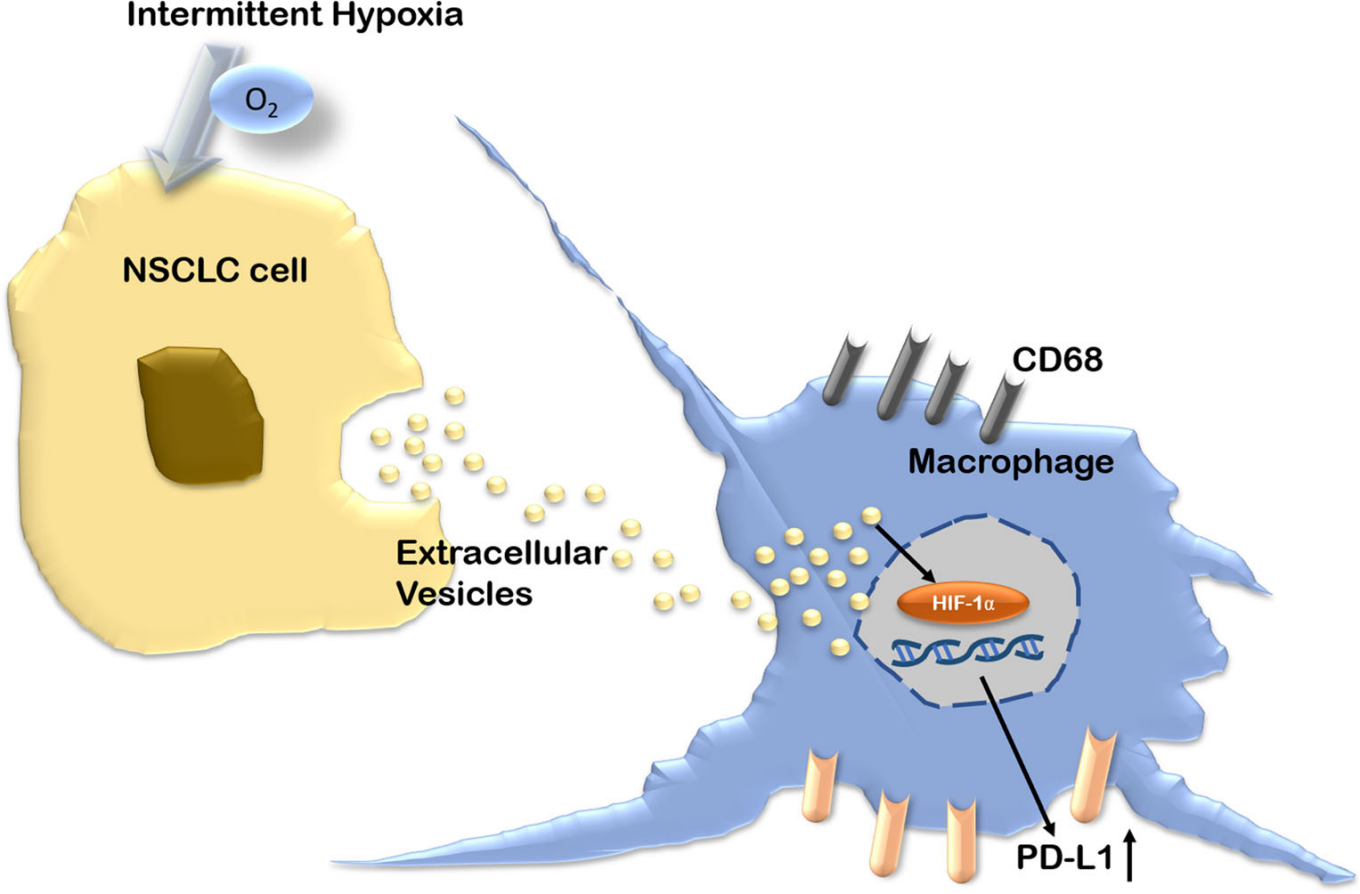
Schematic diagram of IH treated lung cancer cell-derived EVs regulate the function of macrophages. EVs from intermittent hypoxia treated NSCLC cell upregulate PD-L1 expression through HIF-1α pathway in macrophages

To study if HIF-1α is indeed required for the expression of PD-L1 induced by EV-IH, we detected the expression of HIF-1α in macrophages co-cultured with EV-IH or EV-NA. Interestingly, we found a positive relationship between HIF-1α protein levels and PD-L1 expressions. Additionally, the upregulation of PD-L1 expression was reversed by specific HIF-1α inhibitor, which supports the hypothesis that EVs derived from intermittent hypoxic lung cancer cells upregulate PD-L1 in macrophages via HIF-1α (Fig. 5).

This study has several limitations. First, the sample size is limited. A trial with larger sample size is currently being designed to further confirm our findings and investigate the relationship between the effect of EV treatment and immune parameters. Second, we detected the PD-L1 expression on monocytes to approximatively evaluate the immune status of macrophages. Another study is under way to measure the immune status of macrophages in lung cancer tissue from NSCLC patients with and without OSA. Third, we treated EVs as a whole to investigate the biological function, without further analysis the EV cargo. Further research is needed to explore which component in EVs upregulate PD-L1 in macrophages through HIF-1α pathway.

## Conclusion

In this study, we found that PD-L1^+^ monocytes were increased in NSCLC patients with OSA and the expression levels of PD-L1 on monocytes were related to the severity of OSA and nocturnal desaturation. Also, we showed that OSA can enhance the function of tumor-derived EVs to aggravate immunosuppressive status in macrophages. EVs obtained from lung cancer cells treated with IH showed the same effects on macrophages. The possible mechanism was associated with the HIF-1α pathway. This study provides biological plausibility to explain the increased tumor malignancy observed in patients with cancer and OSA.

## Supplementary Information

ESM 1 (DOC 483 kb)

## Data Availability

All data discussed in the manuscript are included within this published article.
