# Supplementary material for: Extracellular vesicles derived from lung cancer cells exposed to intermittent hypoxia upregulate programmed death ligand 1 expression in macrophages
Source: Sleep Breath. 2021 Jul 12;26(2):893–906. doi: 10.1007/s11325-021-02369-1 (PMC9130183; doi:10.1007/s11325-021-02369-1)

# Journal：*sleep and breathing*

# Extracellular Vesicles Derived from Lung Cancer Cells Exposed to Intermittent Hypoxia Up-regulate Programmed Death Ligand 1 Expression in Macrophages

Yuanling Liu ^1,2,3^, Minzhen Lu^1,2,3^,Jianan Chen^2,3^,Siqi Li^1,2,3^,Yiyu Deng^4^,Shifang Yang^2,3^,Qiong Ou^2,3^,Jing Li^2^,Ping Gao^2,3^,Zeru Luo^2,3^,Ping Yuan^2,3^,Jianlong Tan^2,3^,Xinglin Gao^2,3,1^

Author affiliations: ^1^ The Second School of Clinical Medicine, Southern Medical University, Guangzhou, 510515, China. ^2^ Department of Respiratory and Critical Care Medicine, Guangdong Provincial People’s Hospital, Guangdong Academy of Medical Sciences, Guangzhou, 510080, China. ^3^ Guangdong Provincial Geriatrics Institute, Guangzhou, 510080, China. ^4^ Department of Critical Care and Emergency, Guangdong Provincial People’s Hospital, Guangdong Academy of Medical Sciences, Guangzhou, 510080, China.

Correspondence: Xinglin Gao, Ph.D.

Guangzhou, Zhongshan 2^nd^ road No.106, China,510080

Tel +86 13503076147

Fax +86 020-83827812

Email : xinglingao@hotmail.com

**Supplemental Tables**

**Table S1** Clinic characteristics of subjects for exosomes collection.

| Parameter | N  n=4 | N+O  n=4 | *P* vaule |
| --- | --- | --- | --- |
| Age (years) | 70.5(57,75) | 60(54.25,63.75) | 0.200 *^a^* |
| Male proportion | 3(75) | 3(75) | 1.000 ^b^ |
| BMI (kg/m^2^) | 22.75(20.48,24.81) | 26.22(20.78,27.29) | 0.343 *^a^* |
| Smoking proportion | 2(50) | 3(75) | 1.000 ^b^ |
| Medical history |  |  | 1.000 ^b^ |
| Coronary heart disease | 0 | 0 |  |
| Hypertension | 0 | 0 |  |
| Diabetes | 0 | 0 |  |
| Histology |  |  | 1.000 ^b^ |
| Squamous cell carcinoma | 1 | 1 |  |
| Adenocarcinoma | 3 | 3 |  |
| Pathological stage |  |  | 1.000 ^b^ |
| Ⅰ | 1 | 1 |  |
| Ⅱ | 0 | 0 |  |
| Ⅲ | 3 | 3 |  |
| Ⅳ | 0 | 0 |  |
| AHI (events/h) | 2.60(1.65,3.78) | 38.40(22.85,56.88) | 0.029 *^a^* |
| ODI (events/h) | 4.35(0.35,9.03) | 33.35(12.58,68.90) | 0.029 *^a^* |
| ESS | 2.50(0,7.25) | 15.00(9.25,19.25) | 0.029 *^a^* |
| SpO_2_<90% (% TRT) | 0.10(0.03,3.48) | 22.55(10.80,29.88) | 0.029 *^a^* |
| Lowest SpO_2_(%) | 88.00(84.00,94.25) | 67.50(54.50,75.25) | 0.029 *^a^* |
| PD-L1^+^monocytes (%) | 87.05(79.20,93.70) | 95.90(89.38,99.28) | 0.114 *^a^* |
| PD-L1 MFI | 2139.00(2038.50,2234.25) | 4729.50(2746.25,6187.75) | 0.029 *^a^* |

Data are presented as n, n (%) or median (IQR). OSA= obstructive sleep apnea; NSCLC=non-small-cell lung cancer; ESS= Epworth sleepiness scale; AHI= apnea hypopnea index; ODI=oxygen desaturation index; TRT=total recording time; MFI= mean fluorescence intensity

*^a^* Mann-Whitney U tests

^b^ Fisher's exact tests

**Supplemental Figures**

**Fig. S1 Schematic diagram of in vitro IH model.**

A. Hypoxia condition was performed using Modular Incubator Chambers (Billups Rothenberg Inc., San Diego, CA) with an O_2_ Quickstick Oxygen Analyzer (Nuvair, CA93033, USA).


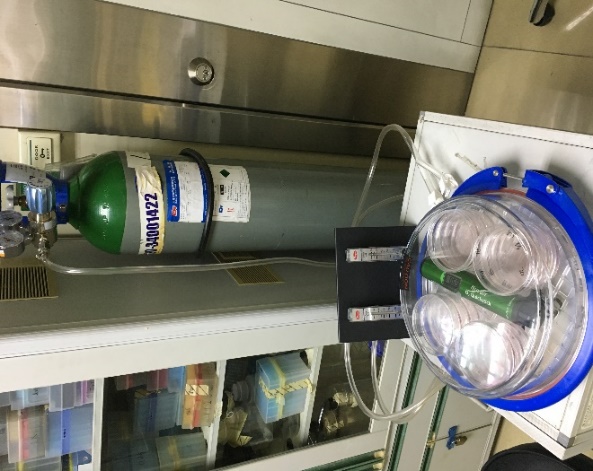


B. IH condition (48 cycles of 5 min of 1% O_2_, followed by 5 min of 21% O_2_).


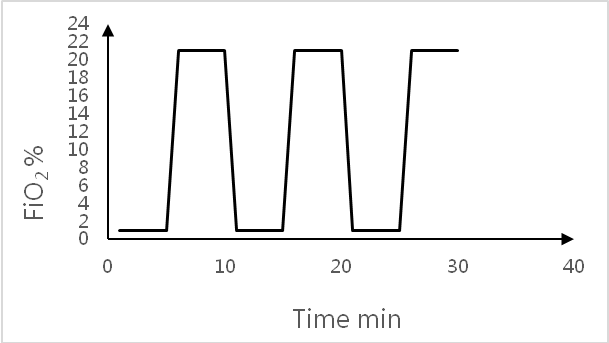


**Fig. S2**

**Phenotypic analysis of CD14^+^PD-L1^+^(A), CD68^+^PD-L1^+^ (B).** Cells were gated according to blank controls or isotype controls.

A.


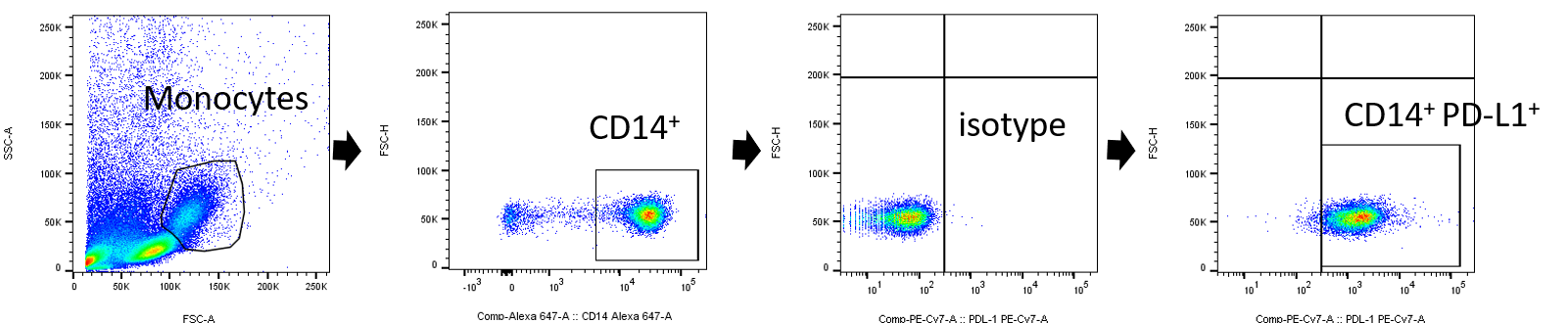


B.
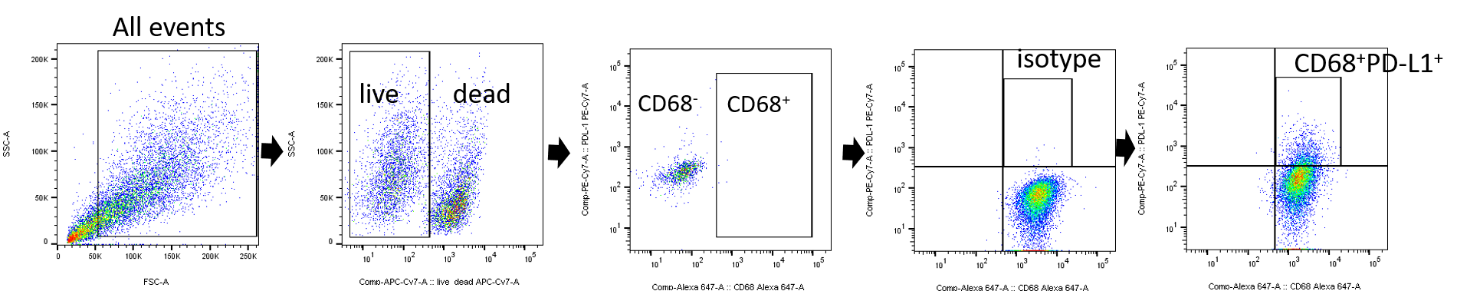

Supplement: Supplementary file 1 — (DOC 483 kb) [file 11325_2021_2369_MOESM1_ESM.doc]
